# Medial abrasion syndrome: a neglected cause of persistent pain after knee arthroplasty

**DOI:** 10.1186/s13018-020-02191-7

**Published:** 2021-01-19

**Authors:** Shaw-Ruey Lyu, Chia-Chen Hsu, Jung-Pin Hung

**Affiliations:** 1 Joint Center, Dalin Tzu Chi General Hospital, Chiayi, Taiwan; 2 Tzu Chi University, Hualien City, Taiwan

**Keywords:** Medial abrasion syndrome, Arthroscopic medial release, Knee arthroplasty, Painful prosthesis, Medial plica, Knee osteoarthritis

## Abstract

**Introduction:**

Persistent post-operative pain (PPOP) has detracted from some otherwise successful knee arthroplasties. This study investigated medial abrasion syndrome (MAS) as a cause of PPOP after knee arthroplasty. The surgical techniques and outcomes of incorporating this concept into the management of both primary arthroplasty cases and patients suffering from unknown causes of PPOP after arthroplasties were presented.

**Materials and methods:**

In a 1-year period, the author performed unicompartmental or total knee arthroplasty (the UKA or TKA group) that also eliminated medial abrasion phenomenon (MAP) on 196 knees of 150 patients at advanced stages of knee osteoarthritis (OA). During the same year, 16 knees of 16 patients with unknown causes of PPOP after knee arthroplasties were referred to the author for the arthroscopic medial release procedure (the AMR group) after being diagnosed as MAS. Subjective satisfaction, Knee Society Score (KSS), and Knee injury and Osteoarthritis Outcome Score (KOOS) evaluations were used for outcome study.

**Results:**

All 166 patients were followed for more than 3 years (mean 3.7 years, 3.1–4.2) for the outcome study. All knees receiving arthroplasty showed medial plicae with MAP at the time of surgery. Only 2 of them suffered from PPOP: one was a neglected tibial plateau fracture with residual varus deformity after UKA, and the other was a late infection after TKA and received revision. The satisfactory rate was 98.8% in the UKA group, 99.1% in the TKA group, and 100% in the AMR group. The Knee Society Scores and all subscales of KOOS were statistically improved in all groups.

**Conclusions:**

MAS is a cause of pain in patients who have received knee arthroplasties, and MAP should be eliminated to ensure a successful knee arthroplasty. PPOP after knee arthroplasty can be caused by MAS, which can be managed by AMR.

**Supplementary Information:**

The online version contains supplementary material available at 10.1186/s13018-020-02191-7.

## Background

Knee arthroplasty has generally been highly successful when judged by prosthesis-related outcomes. However, persistent post-operative pain (PPOP) has been a common occurrence in about 10 to 53% of patients after total knee arthroplasty (TKA) and is associated with reduced health-related quality of life [1–5]. Studies regarding unicompartmental knee arthroplasty (UKA) also reported a high incidence of medial knee discomfort and pain which did not correlate with the post-operative radiographic scores, pre-operative arthritis, and the positioning of the prosthesis and might lead to unnecessary revisions [6–9].

Many factors may be responsible for a painful knee prosthesis. Common causes of prosthetic failure, such as aseptic loosening, infection, instability, progressive patellar arthropathy, and recurrent synovitis, are associated with clearly defined radiographic and/or clinical evidence [10, 11]. Nevertheless, it can be extremely difficult to diagnose and treat a painful knee prosthesis if there is no clear evidence of any of those most common causes of failure. In cases of unexplained pain, reoperation is unwise and frequently associated with suboptimal results [12, 13].

Since medial abrasion phenomenon (MAP)-related medial abrasion syndrome (MAS) is a common cause of knee pain in middle and old age with knee OA [14], its role as a cause of PPOP after a knee arthroplasty cannot be disregarded. We have therefore included the elimination of the MAP in our knee arthroplasty procedures since 2010. In this study, we retrospectively evaluated our case series in a 1-year period for the incidence of PPOP after knee arthroplasties incorporating the technique of MAP elimination. The outcomes of arthroscopic medial release (AMR) [15] for referred patients with unknown causes of PPOP during the same time period were also investigated. We postulate that MAS is a cause of PPOP after knee arthroplasty, and it could be treated with AMR.

## Materials and method

In a 1-year duration (2015), 196 knees of 150 patients at advanced stages of knee OA received arthroplasty (UKA, 80 knees of 66 patients; TKA, 116 knees of 84 patients) by the first author. In the same year, 16 knees of 16 patients with unknown causes of PPOP after knee arthroplasty (UKA, 2 knees; TKA, 14 knees) were referred to our service from other hospitals for AMR management. These patients were prospectively followed as part of an Institutional Review Board Registry. The distribution of age, sex, the main compartment involved, and the type of arthroplasty performed on these knees are listed in Table 1. For the arthroplasty group, the inclusion criteria were advanced primary OA (stage IV or V according to Lyu’s clinical classification [16]). UKA was performed for stage IV or V OA involving only one compartment. For stage IV or V OA involving more than one compartment, TKA was undertaken. All patients who received AMR were referred to our service from other institutions with the diagnosis of PPOP of unknown causes after their knee arthroplasty and had been treated conservatively, including physical therapy and medication, for more than 1 year. Before AMR was performed, evidence of any clearly defined common causes of prosthetic failure including aseptic loosening, instability, progressive patellar arthropathy, infection, and recurrent synovitis was ruled out by radiographic and laboratory examinations. Typical symptoms and signs of pain, crepitus, snapping, localized tenderness, or palpable band described in a previous report [14] confirmed the diagnosis of MAS before surgery.

**Table 1:** Age, sex, and main involved compartment distribution in a different type of surgery *SD* standard deviation, *No.* number, *F* female, *M* male, *Med.* medial compartment, *Lat.* lateral compartment

UKA			TKA			AMR		
Age (SD), No.	F/M (ratio)	Med./Lat. (ratio)	Age (SD), No.	F/M (ratio)	Med./Lat. (ratio)	Age (SD), No.	F/M (ratio)	TKA/UKA (ratio)
72.1 (7.8), 80	53/27 (2.0)	80/0	74.0 (6.6), 116	95/21 (4.5)	104/12 (8.7)	76.3 (5.7), 16	15/1 (15)	14/2 (7)

### Surgical procedures for arthroplasty

**Fig. 1:**
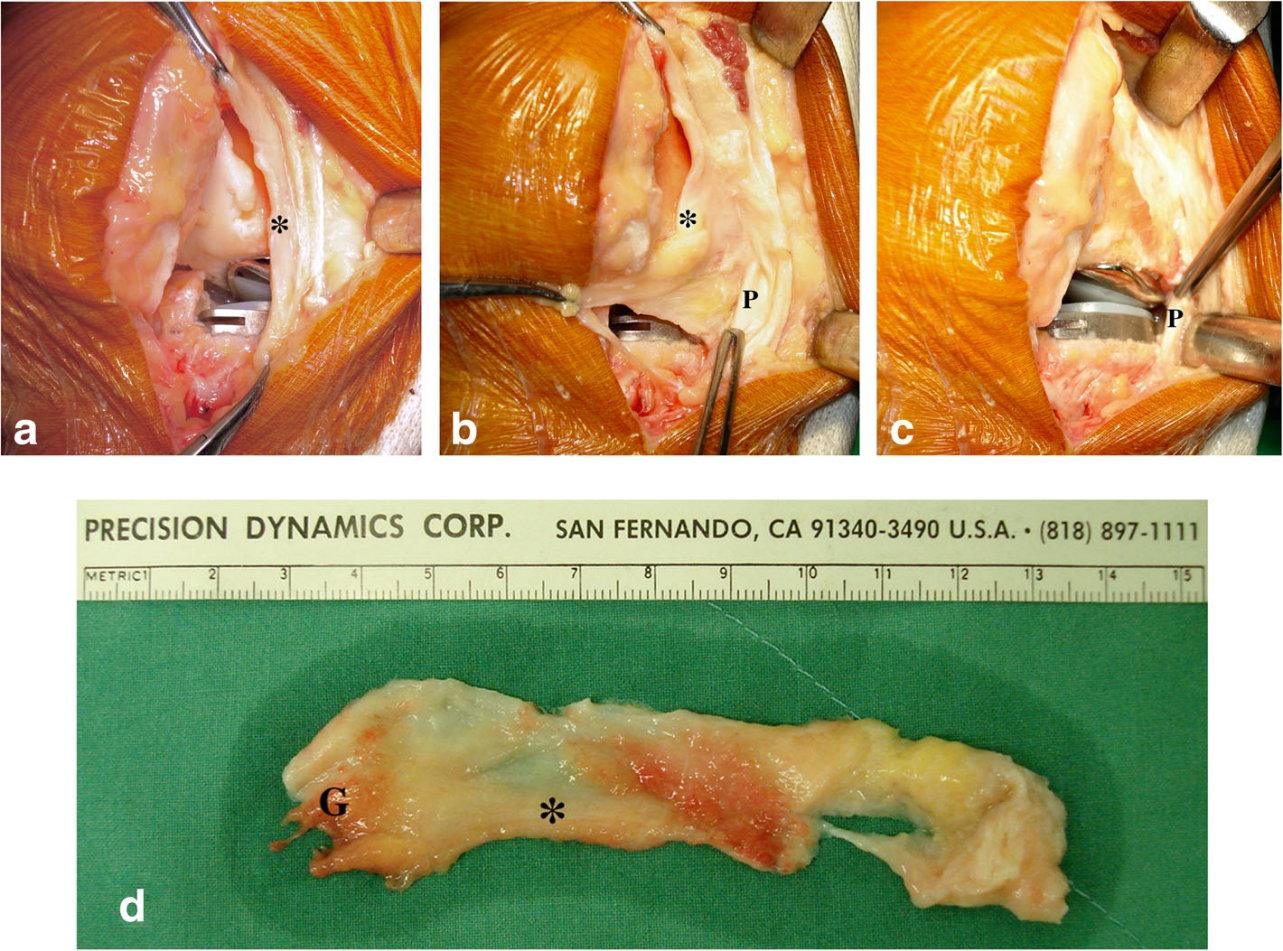
Elimination of medial abrasion phenomenon. **a** Before wound closure, the medial plica (*) was identified in the medial gutter. **b** The synovial fold of the distal part of the medial plica (*) was found to have originated from the tendon sheath of the pes anserinus (P). **c** After the medial plica was completely removed, the prosthesis was clearly visible. **d** Various degrees of wear and fibrillation could be found along the margin of the medial plica, which was always hypertrophied and sometimes became cord-like (*); a small branch of the skeletal muscle originating from the genu articularis (G) was found in all knees


For UKA, all cases were performed with the same cemented, metal-backed fixed bearing implant (ZUK; Zimmer, Warsaw, IN, USA); for TKA, a cemented posterior-stabilized implant (NexGen LPS-flex fixed knee system; Zimmer, Warsaw, IN, USA) was used in all cases. All arthroplasties were performed via a straight anterior incision with a medial parapatellar approach. For UKA, a tibia-first extension gap balancing technique was used. For TKA, the technique utilizing intramedullary femoral and extramedullary tibial alignment guides was followed. After the installation of the prosthesis, the elimination of the existing MAP was performed before wound closure. Various severity of pathologic medial plica described in previous literature [17] could be identified in the medial gutter. As shown in Fig. 1 and Additional file 1: Video 1, the thickened medial plica was removed completely from the attachment of the genu articularis to the tendon sheath of the pes anserinus.

**Additional file 1: Video 1.** Elimination of MAP.

### Surgical procedure for arthroscopic medial release

**Fig. 2:**
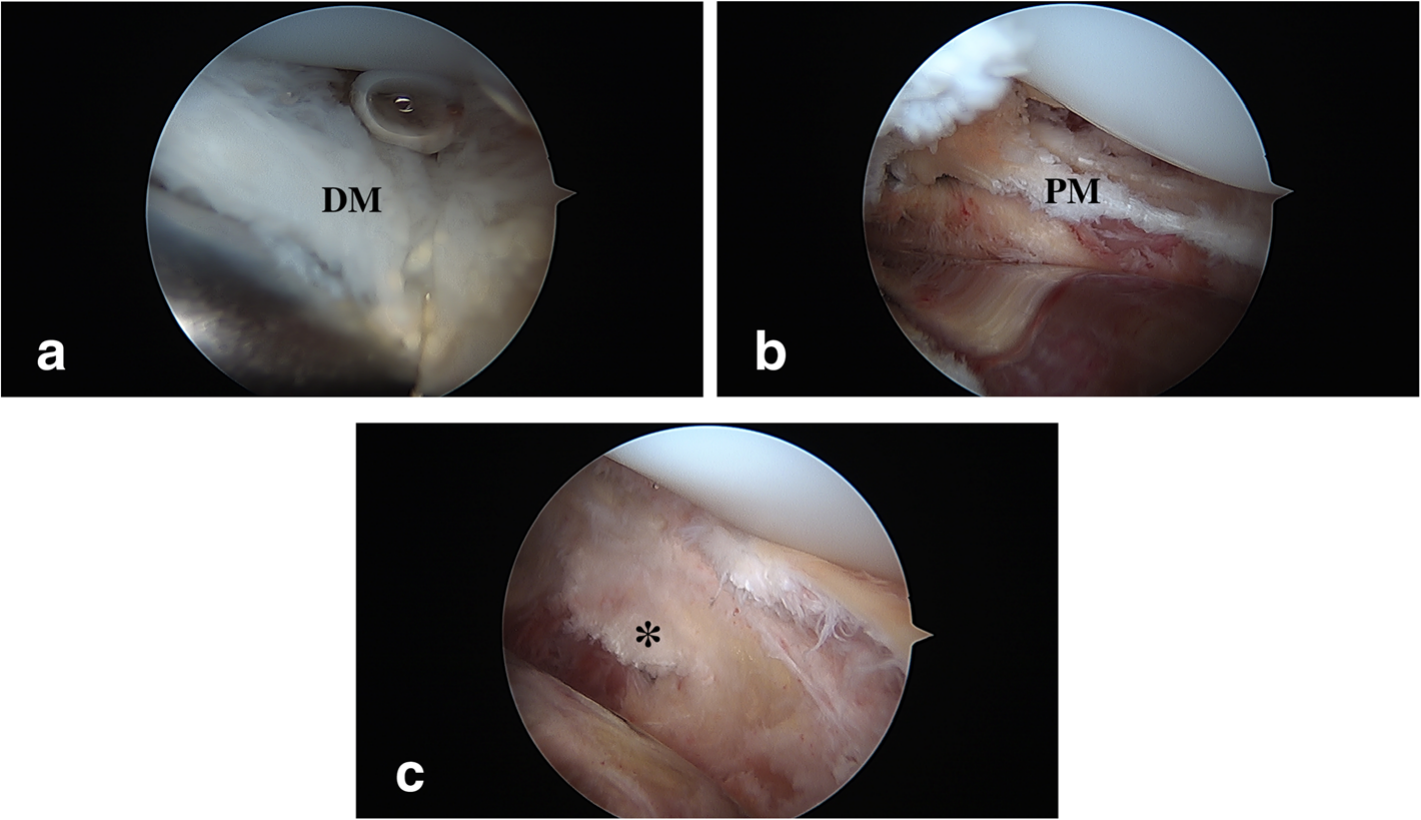
Arthroscopic findings when performing AMR in a patient with PPOP. **a**, **b** Before AMR, the patello-femoral joint was tight, and the fibrotic medial plica was found in the medial gutter (DM, distal medial plica; PM, proximal medial plica). **c** After AMR, the tension of the patello-femoral joint was released, and the medial retinaculum (*) was clearly visible


During arthroscopic examination, remnants or fibrosis of medial plica could be identified over the inferior-medial aspect of the patellofemoral joint (PFJ) as shown in Fig. 2a. Tightness of the PFJ and impingement of the fibrotic medial plica were also verified (Fig. 2b). AMR was then performed as shown in Additional file 2: Video 2. The adequacy of the medial release was checked by pushing the tip of the scope under the patella and verifying if the previously tightly closed medial PFJ space could be easily opened, and the medial retinaculum was clearly visible when the knee was fully extended (Fig. 2c).

**Additional file 2: Video 2.** AMR for MAS in patient with PPOP.

### Post-operative management

The suction drain was used for all patients. Below-knee stockings to prevent thromboembolic disease for both lower limbs were used. Full range of motion and free ambulation were allowed as tolerated. After discharge from the hospital, home exercise programs, including active range of motion (knee hug and knee press) and quadriceps setting, were emphasized.

### Follow-up and evaluation of outcomes

Regular follow-ups were undertaken monthly for 6 months. Thereafter, patients returned yearly for outcome evaluation including clinical and radiographic examinations. The comparisons of both pre- and post-operative Knee Society Score (KSS) and Knee Injury and Osteoarthritis Outcome Score (KOOS) were used for outcome evaluation. Subjective satisfaction was assessed by direct question using a categorical scale prepared for this study: excellent, free of symptoms, no limitation in activities; good, greatly improved, occasional pain, normal activities; fair, same as pre-operative condition, no improvement; and poor, has received or considered further operative treatment. The outcome was regarded as satisfactory if subjective satisfaction was rated as “excellent” or “good.” The inquiry into subjective satisfaction and the evaluation of KSS and KOOS were conducted by nursing specialists. All investigations focused on individual knees in bilateral cases.

### Statistical evaluation

All values were presented with means and standard deviations. Statistical analysis for comparing pre-operative and post-operative KSS and KOOS was performed using the paired *t* test. *p* < 0.05 was considered to be statistically significant. All statistical analysis was carried out using JMP, the Statistical Discovery Software (version 5.0.1.2, SAS Institute Inc., Cary, NC, USA).

## Results

All patients were successfully followed for more than 3 years (mean, 3.7 years, 3.1–4.2) and were available for the outcome study including subjective satisfaction, KSS, and KOOS evaluations. Medial plicae with MAP were identified in all the knees that received arthroplasty. Two patients suffered from PPOP: one patient in the UKA group had persistent pain due to a neglected tibial plateau fracture with residual varus deformity; the only dissatisfied patient in the TKA group suffered from a late infection with persistent pain and received revision. The satisfactory rate was 98.8% in the UKA group, 99.1% in the TKA group, and 100% in the AMR group (Table 2). The Knee Society Scores and all subscales of KOOS were statistically improved in all groups (Tables 3 and 4). No evidence of loosening or wearing of the prostheses was found by radiographic examinations in all groups.

**Table 2:** Subjective outcomes stratified by type of surgery

Operation (*N*)	E	G	F	P	Sat. (%)
AMR (16)	10 (62.5)	6 (37.5)	0	0	100.0
UKA (80)	74 (92.5)	5 (6.3)	0	1 (1.2)	98.8
TKA (116)	108 (93.1)	7 (6.0)	0	1 (0.9)	99.1

*E* excellent, *G* good, *F* fair, *P* poor, *Sat.* satisfied = E + G

**Table 3:** Pre-operative and post-operative Knee Society Score for different operation

Operation (*N*)	Pain (SD)		Function (SD)	
	Pre-op.	Post-op.	Pre-op.	Post-op.
AMR (16)	55.4 (11.5)	90.6 (9.3)	47.8 (15.8)	79.4 (14.7)
Uni-K (80)	60.7 (13.6)	92.7 (9.0)	46.2 (17.3)	82.9 (15.2)
TKA (116)	53.1 (19.6)	95.8 (5.8)	38.2 (20.0)	80.2 (16.8)
*p* value	< 0.001		< 0.001

**Table 4:** Pre-operative and post-operative KOOS for different operation

Operation (*N*)	P		S		ADL		S/R		QOL	
	Pre-op. (SD)	Post-op. (SD)	Pre-op. (SD)	Post-op. (SD)	Pre-op. (SD)	Post-op. (SD)	Pre-op. (SD)	Post-op. (SD)	Pre-op. (SD)	Post-op. (SD)
AMR (16)	54.3 (14.4)	93.7 (7.3)	46.6 (15.9)	77.7 (15.2)	63.8 (9.4)	89.4 (10.5)	16.1 (18.6)	38.4 (31.2)	34.0 (19.2)	66.0 (24.7)
Uni-K (80)	53.3 (18.1)	93.7 (10.4)	47.8 (15.2)	86.9 (15.4)	60.2 (18.4)	89.5 (16.7)	22.8 (21.4)	61.9 (35.3)	37.7 (18.4)	82.1 (21.4)
TKA (116)	50.5 (17.7)	95.9 (7.2)	46.1 (15.1)	89.0 (12.3)	56.3 (19.3)	93.9 (8.3)	22.7 (21.2)	60.4 (30.7)	33.1 (16.7)	84.1 (20.4)
*p* value	< 0.001		< 0.001		< 0.001		< 0.001		< 0.001	

*P* pain, *S* other symptoms, *ADL* function in activity of daily living, *S/R* function in sport and recreation, *QOL* knee-related quality of life

## Discussion

PPOP during everyday activities or at rest is a major cause of patient dissatisfaction after knee arthroplasty. It affects the quality of life of a significant percentage of patients who have undergone TKA [1–5, 18] and is also a common post-operative complaint after UKA [6–9]. According to the findings of this study, the elimination of MAP during either TKA or UKA could avoid unknown causes of PPOP during more than 3 years of follow-up. Moreover, patients with unknown causes of PPOP after arthroplasties have become satisfied after their MAP were treated with AMR.

Many theories have been proposed to explain the etiology and risk factors of PPOP after knee arthroplasty [19, 20]. But, despite medical advances, unknown causes of PPOP has remained a clinical problem, and it is not clear why these conditions occur. It can be described as retropatellar or peripatellar pain, which limits patients in their everyday activities. Patients might experience difficulty in standing up from a chair, walking up and down the stairs, and riding a bicycle. Sometimes, even trying to put their knees in an extension position is painful and difficult. This troublesome situation remains a challenge for the surgeons and usually leads to two questions: “How can a ‘perfectly’ placed knee arthroplasty (TKA or UKA) still be painful?” and “What may have caused this pain?” [19]. Some patients might therefore be claimed to have a higher than normal depressive or anxiety state [20].

To increase overall patient satisfaction, it is important to identify the different anatomical structures that can cause this pain so as to prevent PPOP after knee arthroplasty. MAS has been reported to be a neglected cause of knee pain in middle and old age with knee OA, and it can be effectively treated with AMR [14]. Incorporation of the elimination of MAP in arthroplasty has significantly decreased the incidence of PPOP in this series compared to previous literatures [1–9, 18]. For referred cases with unknown causes of PPOP, we also found that AMR could deliver satisfactory outcomes for the patients. Both of these findings have shone light on the important role that MAP plays in causing pain after arthroplasty.

Arthroscopy for failed knee arthroplasty is a well-documented and accepted procedure for the diagnosis of component and soft tissue problems [10, 21–23]. It is a valuable tool to evaluate a painful knee arthroplasty, and it can be used to treat certain conditions such as removing loose bodies, correcting patella subluxation with a lateral release, excising a symptomatic pseudomeniscus, and releasing intraarticular adhesions to improve motion and relieve pain that would otherwise require an arthrotomy [24]. However, MAS and AMR have not been reported in the literature regarding PPOP. Our study has broadened the therapeutic value of arthroscopy for patients with PPOP.

There are some limitations to this study. First, it is a retrospective study of a consecutive case series, and there was no comparative group. Nevertheless, the significant low incidence of PPOP after arthroplasty incorporating elimination of MAP in this series compared to other reports [1–9] still provides evidence to our hypothesis that MAS is a cause of PPOP. Second, as this is a case series by a single surgeon who utilized the same surgical technique in every case, we are unable to assess other factors such as the approaches and the extent of fat pad resection which might be associated with PPOP after arthroplasty. Third, the low number of patients who received AMR for PPOP with unknown causes may have overemphasized the incidence of MAS as a cause of PPOP. However, these drawbacks might draw attention to further investigations.

## Conclusion

MAS is a neglected cause of knee pain in patients who have received arthroplasties. Elimination of MAP during knee arthroplasty could lower the incidence of PPOP. Unrecognized and untreated MAP in arthroplasty is a cause of PPOP, and it can be managed by AMR.

## Data Availability

The datasets during and/or analyzed during the current study are available from the corresponding author on reasonable request.

## Abbreviations

PPOP: Persistent post-operative pain
MAS: Medial abrasion syndrome
AMR: Arthroscopic medial release
UKA: Unicompartmental knee arthroplasty
TKA: Total knee arthroplasty
OA: Osteoarthritis
KSS: Knee Society Score
KOOS: Knee Injury and Osteoarthritis Outcome Score
MAP: Medial abrasion phenomenon
PFJ: Patellofemoral joint

## References

[1] Beswick AD, Wylde V, Gooberman-Hill R, Blom A, Dieppe P (2012). What proportion of patients report long-term pain after total hip or knee replacement for osteoarthritis? A systematic review of prospective studies in unselected patients. BMJ Open.

[2] Liu SS (2012). A cross-sectional survey on prevalence and risk factors for persistent postsurgical pain 1 year after total hip and knee replacement. Reg Anesth Pain Med.

[3] Skou ST, Graven-Nielsen T, Rasmussen S, Simonsen OH, Laursen MB, Arendt-Nielsen L. Facilitation of pain sensitization in knee osteoarthritis and persistent post-operative pain: a cross-sectional study. Eur J Pain. 2014;18(7):1024–31. 10.1002/j.1532-2149.2013.00447.x. 10.1002/j.1532-2149.2013.00447.x 24375931

[4] Bourne RB, Chesworth BM, Davis AM, Mahomed NN, Charron KDJ (2010). Patient satisfaction after total knee arthroplasty: who is satisfied and who is not?. Clinical Orthopaedics and Related Research.

[5] Breugem SJ, van Ooij B, Haverkamp D, Sierevelt IN, van Dijk CN. No difference in anterior knee pain between a fixed and a mobile posterior stabilized total knee arthroplasty after 7.9 years. Knee Surg Sports Traumatol Arthrosc. 2014;22(3):509–16. 10.1007/s00167-012-2281-2. 10.1007/s00167-012-2281-2 23124601

[6] Edmondson MC, Isaac D, Wijeratna M, Brink S, Gibb P, Skinner P. Oxford unicompartmental knee arthroplasty: medial pain and functional outcome inthe medium term. J Orthop Surg Res. 2011;6:52. 10.1186/1749-799X-6-52Ref>. 10.1186/1749-799X-6-52 PMC3198686 21981987

[7] Baker PN, Petheram T, Avery PJ, Gregg PJ, Deehan DJ. Revision for unexplained pain following unicompartmental and total knee replacement. J Bone Joint Surg Am. 2012;94(17):e126. 10.2106/JBJS.K.00791. 10.2106/JBJS.K.00791 22992855

[8] Crawford DA, Berend KR, Lombardi AV. Management of the Failed Medial Unicompartmental Knee Arthroplasty. J Am Acad Orthop Surg. 2018;26(20):e426–e433. 10.5435/JAAOS-D-17-00107. 10.5435/JAAOS-D-17-00107 30113345

[9] Crawford DA, Berend KR, Lombardi AV. Management of the Failed Medial Unicompartmental Knee Arthroplasty. J Am Acad Orthop Surg. 2018;26(20):e426–e433. 10.1007/s00167-020-05861-5. 10.5435/JAAOS-D-17-00107 30113345

[10] Klinger H-M, Baums MH, Spahn G, Ernstberger T (2005). A study of effectiveness of knee arthroscopy after knee arthroplasty. Arthroscopy.

[11] Villano M, Carulli C, Puccini S, Soderi S, Innocenti M (2011). Painful knee prosthesis: surgical approach. Clin Cases Miner Bone Metab.

[12] Dennis DA (2004). Evaluation of painful total knee arthroplasty. J Arthroplast.

[13] Toms AD, Mandalia V, Haigh R, Hopwood B (2009). The management of patients with painful total knee replacement. J Bone Joint Surg Br..

[14] Lyu S-R, Lee C-C, Hsu C-C. Medial abrasion syndrome: a neglected cause of knee pain in middle and old age. Medicine (United States). 2015;94(16). 10.1097/MD.0000000000000736. 10.1097/MD.0000000000000736 PMC4602696 25906102

[15] Lyu SR, Lee CC, Hsu CC. Medial abrasion syndrome: a neglected cause of knee pain in middle and old age. Medicine (Baltimore). 2015;94(16):e736. https://doi.org/10.1097/MD.0000000000000736. 10.1097/MD.0000000000000736 PMC4602696 25906102

[16] Grosu I, Lavand'homme P, Thienpont E. Pain after knee arthroplasty: an unresolved issue. Knee Surg Sports Traumatol Arthrosc. 2014 Aug;22(8):1744–58. 10.1007/s00167-013-2750-2. 10.1007/s00167-013-2750-2 24201900

[17] Lyu SR, Chiang JK, Tseng CE. Medial plica in patients with knee osteoarthritis: a histomorphological study. Knee Surg Sports Traumatol Arthrosc. 2010;18(6):769–76. 10.1007/s00167-009-0946-2. 10.1007/s00167-009-0946-2 19826785

[18] Grosu I, Lavand'homme P, Thienpont E. Pain after knee arthroplasty: an unresolved issue. Knee Surg Sports Traumatol Arthrosc. 2014 Aug;22(8):1744–58. 10.1007/s00167-013-2750-2. 10.1007/s00167-013-2750-2 24201900

[19] Breugem SJ, Haverkamp D. Anterior knee pain after a total knee arthroplasty: What can cause this pain?. World J Orthop. 2014;5(3):163-170. 10.5312/wjo.v5.i3.163. 10.5312/wjo.v5.i3.163 PMC4095008 25035818

[20] Bonnin, M.P., Basiglini, L. & Archbold, H.A.P. What are the factors of residual pain after uncomplicated TKA?. Knee Surg Sports Traumatol Arthrosc. 2011;19:141–17. 10.1007/s00167-011-1549-2. 10.1007/s00167-011-1549-2 21598009

[21] Bocell JR, Thorpe CD, Tullos HS. Arthroscopic treatment of symptomatic total knee arthroplasty. Clin Orthop Relat Res. 1991;(271):125–34. 1914287

[22] Wasilewski SA, Frankl U (1989). Arthroscopy of the painful dysfunctional total knee replacement. Arthroscopy.

[23] Wong JWK, Yau PWP, Chiu PKY (2002). Arthroscopic treatment of patellar symptoms in posterior stabilized total knee replacement. Int Orthop.

[24] Johnson DR, Friedman RJ, McGinty JB, Mason JL, Mary EWS (1990). The role of arthroscopy in the problem total knee replacement. Arthroscopy.

